# Cancer in Croatia; where do we stand and how to move forward?

**DOI:** 10.3325/cmj.2012.53.91

**Published:** 2012-04

**Authors:** Eduard Vrdoljak

**Affiliations:** Center of Oncology, University Hospital Split, Medical School Split, Split, Croatia

Cancer is one of the leading causes of morbidity and mortality in the world, and the global burden of cancer is predicted to grow rapidly in the coming decade. While cancer affects all communities indiscriminately, there are striking differences in the incidence and mortality among different populations. These large differences reflect a combination of differences in the prevalence of risk factors, differences in genetic susceptibility, and/or variations in cancer detection, reporting, classification systems, treatment, and follow-up. Unfortunately, developing nations or nations in the so called economical transition are those with the fastest growing cancer problem. The International Agency for Research on Cancer estimated that over a half of newly diagnosed cases and two-thirds of cancer deaths occur in low and medium-income countries (1). Priority setting for cancer control and cancer services in any region needs to be based on knowledge of the cancer burden and of the local mix of predominant cancer types (2).

There are striking variations in the regional cancer patterns. Among European countries, wide differences are observed in the quality of cancer care, especially between developed and low-resource, transitional countries (3). In general, cancer survival is significantly lower in transitional European countries than in the developed ones (3).

In order to appropriately allocate financial and other resources in the fight against cancer it is essential to have epidemiological information, plan treatment strategies, and address access issues. An important first step that could provide a basis on which to plan future epidemiology projects and ultimately to improve the quality of cancer care in Croatia is gaining accurate knowledge on the cancer registry data in Croatia.

This issue of the *Croatian Medical Journal* (CMJ) publishes six articles by different groups of authors led by Dr Ariana Znaor from Croatian National Institute of Public Health (4-9). They analyzed melanoma, lung, colon, gastric, breast, uterine, ovarian, cervical, prostate cancer, and leukemia and lymphoma incidence and mortality trends in Croatia from 1988 till 2008, and compared them with the trends in other, especially European populations. Their epidemiological analysis indicates the scale of the problem of oncological care in Croatia. Also, the comparison of the oncology status between Croatia and other countries is of great importance since the key barriers to delivery of appropriate quality of cancer care within limited resources environment have not been previously identified. Unfortunately, incidence and mortality trends for most of investigated cancer sites were less favorable than in Western European countries (10,11). In particular, Croatia has one of the highest rates in Europe for lung cancer and other tobacco-related cancers (5). Of course, it is well known that to have favorable trends in cancer mortality, societies would require a strategy focusing on the control of tobacco and alcohol consumption, adequate nutrition, and avoidance of excessive sun exposure (12). Early diagnosis can also have a relevant impact, and this together with the universal adoption of recent therapeutic advances and better organization of oncology system in general (adherence to guidelines, referral centers with multidisciplinary team work, optimal access to treatment, especially targeted drugs), may contribute to reducing the cancer mortality burden (10,12).

The most important factors that influence cancer mortality rates are adequate cancer prevention, screening, diagnosis, and treatment (10). Also, the distribution of different types of cancer is equally important. Namely, while in developed countries the most common type of cancer in men is prostate cancer, in the developing world and Croatia the most prevalent cancer is lung cancer, a significantly more dangerous tumor type. The excess mortality from these neoplasms in Croatia and other transitional countries could therefore be reduced if adequate resources, training, and logistics to deliver adequate preventive measures, diagnosis, and treatment were implemented.

In the articles published in this issue of the *CMJ,* the incidence data were obtained from the Croatian National Cancer Registry and the mortality data from the Croatian Bureau of Statistics or from the World Health Organization mortality database. In addition to incidence and mortality data collected by cancer registries, data related to underlying risk factors and treatment of patients should become more available, leading to more important and valuable information from a health outcomes point of view.

In conclusion, new and comprehensive data about selective cancer incidence and mortality in Croatia highlight the importance of the subject and the need to invest more in all known activities related to the fight against cancer – education, prevention, early detection, better access to adequate anticancer treatments, and multidisciplinary work.
